# Extracorporeal Membrane Oxygenation (ECMO): A Life Saver in Near-Fatal Asthma

**DOI:** 10.7759/cureus.20117

**Published:** 2021-12-02

**Authors:** Nouraldeen Manasrah, Basel Abdelazeem, Sarah Al Qasem, Emad Kandah, Ahmed Jamal Chaudhary

**Affiliations:** 1 Internal Medicine, Detroit Medical Center (DMC) Sinai Grace Hospital, Detroit, USA; 2 Internal Medicine, McLaren Health Care/Michigan State University (MSU), Flint, USA; 3 Internal Medicine, Luzmila Hospital, Amman, JOR

**Keywords:** abg, near fatal asthma, invasive mechanical ventilation, asthma exacerbations, vv ecmo

## Abstract

Near-fatal asthma (NFA) is a life-threatening condition that represents the most severe clinical phenotype of asthma and can progress to fatal asthma. Patients with NFA do not respond adequately to conventional medical therapy and urgent intervention is needed to provide adequate oxygenation by invasive mechanical ventilation. While mechanical ventilation is a potentially life-saving intervention, it could cause lung injury, barotrauma, and dynamic hyperinflation due to high ventilator settings resulting in hemodynamic instability. Extracorporeal membrane oxygenation (ECMO) provides full respiratory support with adequate gas exchange in patients with NFA and improves survival rate. We present a case of a young female patient who presented with NFA, and her clinical condition was worsening despite invasive positive pressure mechanical ventilation.

## Introduction

Near-fatal asthma (NFA) is a life-threatening condition associated with hypoxemia, hypercapnia, and altered mental status which may lead to respiratory arrest and requires mechanical ventilation [1]. It is associated with significant morbidity and mortality. Systemic corticosteroids, bronchodilators, and protective mechanical ventilation are the mainstay of treatment for NFA. Other therapies, such as magnesium sulfate, neuromuscular blockade, and helium-oxygen mixtures may have a potential therapeutic role [2]. Due to advances in medical treatment, extracorporeal membrane oxygenation (ECMO) is a promising therapy in NFA [3,4].

## Case presentation

A 42-year-old female with a past medical history of asthma and hypertension was brought to the emergency department because of shortness of breath (SOB). The patient ran out of her asthma medications for three days and started feeling SOB for two days that got worse with time. She denied chest pain, cough, fever, chills, history of blood clots, calf pain, or lower extremity swelling. The patient has never been intubated for asthma. The patient was tachypneic, diaphoretic, and using accessory muscles, speaking in 4-5-word sentences. Vital signs were blood pressure (141/90 mmHg), heart rate (120 beats/minutes), respiratory rate (32 breaths/min), temperature (37.1 celsius), and oxygen saturation was 60% on room air and it improved to 96% on 15L non-rebreather oxygen mask. Cardiovascular examination revealed increased heart rate with a regular rhythm, normal S1, S2, with no murmurs. Respiratory examination revealed decreased breath sounds and wheezing bilaterally.

Complete blood count and basic metabolic panel were within normal range. An electrocardiogram (ECG) showed normal sinus rhythm with no ischemic changes, and a chest x-ray showed no evidence of acute cardiopulmonary processes. The patient was started on intravenous fluid (Normal saline 100cc/h), ipratropium bromide/albuterol nebulizer, intravenous methylprednisolone 80mg, and magnesium sulfate 2g. After receiving breathing treatments, the patient was still having difficulty breathing, so she was placed on bi-level positive airway pressure (BiPAP) with settings of (inspiratory positive airway pressure [IPAP] 10 and expiratory positive airway pressure [EPAP] 5, FiO_2_ of 100%) to reduce the work of breathing. Continuous ipratropium bromide/albuterol was administered through the BiPAP. She was watched closely while on BiPAP for about 20 minutes. Arterial blood gas (ABG) showed severe respiratory acidosis with high serum lactate level (Table 1). The decision was made to intubate the patient due to her severe acidosis and hypercapnia, then the patient was transferred to the intensive care unit (ICU). Ventilatory settings were (assist control mode, respiratory rate of 14, tidal volume 5mL/kg, FiO_2_ 100%, I: E ratio of 1:4, and positive end-expiratory pressure [PEEP] 5 cm H_2_O). She was heavily sedated with propofol and fentanyl drips also, she was paralyzed with IV cisatracurium and started on terbutaline infusion at 0.1 mCg/kg/min. Despite the above treatments and ventilator manipulations to decrease minute ventilation and allow permissive hypercapnia, the peak and plateau airway pressure were persistently high at 57 and 48 cm H_2_O, respectively. Repeat ABG was worse (Table 1). The decision was made to start the patient on Extracorporeal Membrane Oxygenating (ECMO) via the right intrajugular veno-venous route with heparin infusion. The patient was started on ketamine infusion, continued on nebulization, steroids, fentanyl, and heparin infusions. ABG improved over the next three hours (Table 1).

**Table 1:** Serial arterial blood gases BiPAP - Bi-level positive airway pressure; ECMO - Extracorporeal membrane oxygenation

	On admission while on BiPAP	Post mechanical ventilation	After ECMO
FiO_2_ (%)	100	100	80
pH	7.09	7.02	7.38
PaO_2_ (mmHg)	365	216	294
PaCO_2_ (mmHg)	88	98	24.6
HCO_3_ (mmol/L)	27	25	42
Lactate (mmol/L)	2.1	3.2	1.2

Trials for gradually weaning the patient off the ventilator were made until she was successfully extubated after three days of hospitalization, and ECMO was discontinued on the fourth day. The patient was doing well and feeling better. She was transferred to the medical ward after five days stay in the ICU, then she was discharged home after two days in the medical ward.

## Discussion

More than two million emergency department visits and 5,000 to 6,000 deaths have been reported in the United States annually due to asthma exacerbation [5]. NFA has two phenotypes, the first one is characterized by gradual clinical deterioration over days or weeks that usually occurs in patients with severe and poorly controlled asthma, and it is the most common type that represents 85% of cases [1,6]. This type could be preventable with appropriate management and compliance with medications. The second type is more severe, and respiratory failure may develop within a few hours of the onset of symptoms. It occurs in patients who have extreme allergic responses as well as emotional distress [7].

Respiratory acidosis is an ominous finding in severe asthma exacerbation pointing toward severe airway obstruction leading to retention of CO_2_ (gas-trapping) and the development of hypercapnia. Hypoxemia develops due to ventilation-perfusion mismatch and hypoventilation [8]. Patients with NFA do not respond to conventional medical therapy like inhaled β2 adrenergic agonists, anticholinergics, theophylline, epinephrine, and corticosteroids. Invasive mechanical ventilation may be life-saving. Nevertheless, mechanical ventilation could be associated with increased morbidity and mortality in patients with asthma secondary to excessive dynamic pulmonary hyperinflation (DH) with intrinsic positive end-expiratory pressure (intrinsic PEEP or auto-PEEP), which may result in barotrauma [9]. To avoid barotrauma during mechanical ventilation in NFA, permissive hypercapnia should be allowed by a reduction in inspiratory flow rates and tidal volume, which reduces dynamic hyperinflation [10]. Once airway resistance decreases and hypoxemia is corrected with adequate ventilation and medical treatment, hypercapnia recovers shortly. However, in severe cases with extreme airway obstruction, hypoxemia cannot be corrected despite optimal mechanical ventilator use, therefore death can occur due to serve hypoxia.

ECMO can provide efficient gas exchange during acute respiratory failure and help prevent lung injury induced by aggressive mechanical ventilation. A retrospective cohort study of 272 patients with NFA who were started on ECMO reported that ECMO significantly improved gas exchanges and enhanced survival rate more than other respiratory conditions [3]. It can also lower positive inspiratory pressure (PIP), which may reduce ventilator-induced lung injury and oxygen toxicity, and reduce tidal volume and minute ventilation, consequently mitigating dynamic hyperinflation.

Despite the potential benefits of ECMO, complications may limit its use. Few complications can occur during cannulation, including vessel perforation, arterial dissection, and distal ischemia. Other complications include renal failure, pneumonia or sepsis, thrombosis, and bleeding [11].

Therefore, ECMO may be considered in patients with severe hypoxemia, respiratory acidosis, and hemodynamic instability, despite maximal mechanical ventilation settings. Further studies are needed to guide the use of ECMO in NFA.

## Conclusions

NFA is a life-threatening condition that results in profound hypoxemia, hypercapnia, and altered mental status. This case highlights the importance of using ECMO as a rescue therapy in NFA when the highest conventional treatment fails, including invasive mechanical ventilation. The early introduction of ECMO can provide adequate gas exchange, promptly improve respiratory symptoms, overcome ventilator-induced lung injury and oxygen toxicity, and improve survival rate in NFA.
